# 

**Published:** 1906-09-29

**Authors:** 


### BOOKS RECEIVED.

W. B. CLIVE.
"Human Physiology." By G. Norman Meachen, M.D.

H. J. GLAISHER.
"Lectures on Neurasthenia." By T. D. Savill, M.D.

SMITH, ELDER AND CO.
"Artificial Feeding and Food Disorders of Infants." 6th edition. By W. B. Cheadle, M.D.

DAVOS PRINTING CO., LIMITED.
"Davos as Health Resort."

### MEDICAL APPOINTMENTS.

BURFIELD, J., M.B., B.S.Lond., F.R.C.S.Eng., has been appointed Assistant Medical Officer at the Bolingbroke Hospital, Wandsworth Common.

Nearly forty years ago we had considerable experience of the ignorance of Birmingham mothers and did something towards educating them in the matter of the feeding of children. It is interesting to note in this connection that the medical officer of health for Birmingham, in his report of 1905, makes a great point of the large number of deaths due to carelessness and ignorance, especially in relation to the feeding and rearing of children—155 per 1,000 children born dying from this cause. Birmingham was the "mother" of Mr. Forster's Education Act and if the thirty-six years of improved education have left the ignorance of Birmingham mothers untouched it it time that new methods of instruction should be created and enforced.

From investigations made by Dr. H. Wright Thompson into the eyesight of the school population of Glasgow it would appear that more than one out of every three children had defective eyesight. Seeing the evils which arise from the absence of treatment of ophthalmic defects in their early stages, it is desirable that school authorities should take steps to have the eyes of the children attending public schools periodically examined by an ophthalmic surgeon.

### NOTICE TO CORRESPONDENTS.

All MSS., Letters, Books for Review, and other matters intended for the Editor should be addressed The Editor, "The Hospital," The Hospital Buildings, 28 & 29 Southampton Street, Strand, W.C.

The Editor cannot undertake to return rejected MS., even when accompanied by stamped directed envelope.

### Notice to Advertisers and Subscribers.

All Advertisements, Orders for Copies of The Hospital, and Business Communications should be addressed to **THE MANAGER** (**Not to the Editor**), The Hospital, 28 & 29 Southampton Street, Strand London, W.C.

The Cost of Subscription, post free, is as follows:—Per week, 3½d.; per quarter, 3s. 3d.; per half-year, 6s. 6d.; per annum, 13s. Foreign and Colonial:—Per annum, 19s. 6d., payable, in all cases, in advance.

**NOTICE.—Vol. XXXIX. of "The Hospital" (October 1905 to March 1906), in handsome cloth binding, will shortly be on sale at the office of this Journal, price 10s. 6d. Cases for binding the Numbers contained in this Volume 2s. Index to Vol. XXXIX., 3½d., post free.**

**The Monthly Part for September is now ready. Price 1s., by post, 1s. 3d.**

### Two Indispensable Handbooks for Nurses

JUST READY. NEW AND REVISED EDITION.

### SURGICAL INSTRUMENTS AND APPLIANCES.

1/6 net, 1/8 post free.

An Illustrated and Classified List, with Explanatory Notes.

CONTAINING A NEW CHAPTER ON THE CARE AND STERILIZATION OF SURGICAL INSTRUMENTS.

BY

HAROLD BURROWS, M.B.(Lond.), B.S., F.R.C.S.

### THE NURSES' PRONOUNCING DICTIONARY OF MEDICAL TERMS AND NURSING TREATMENT.

2/- post free.

Compiled by HONNOR MORTEN.

NEW EDITION. Revised and Edited by MARY I. BURDETT.

Write for Catalogue of Nursing Manuals, which will be sent post free on application.

**The Scientific Press Ltd.,**

Publishers of Nursing Text-Books, Charts, and Case-Books of all Descriptions.

28 & 29 SOUTHAMPTON STREET, STRAND, LONDON, W.C.

